# Increased serum leptin levels are associated with metabolic syndrome and semen parameters in patients with infertility

**DOI:** 10.1097/MD.0000000000040353

**Published:** 2024-11-01

**Authors:** Zhen Dong, Xin Song, Deping Dong, Zhikang Cai

**Affiliations:** a Department of Urology, Hai’an People’s Hospital, Hai’an, Jiangsu, China; b Department of Urology, Gongli Hospital of Shanghai Pudong New Area, Shanghai, China.

**Keywords:** infertility, leptin, metabolic syndrome, semen parameters

## Abstract

This study aims to ascertain the associations between serum leptin levels and metabolic syndrome and semen parameters in patients with infertility. A total of 200 patients who were diagnosed as primary infertility in our hospital were enrolled in this study, and they were divided into MetS group and non-MetS group. About 30 healthy men were enrolled as the control group. The general information, blood lipids, reproductive hormones and semen parameters were collected. We used the Student *t* test, the Chi-square test, the Kruskal–Wallis test, and the spearman correlation analysis to analyze their relationships. BMI, glucose, TG, follicle-stimulating hormone (FSH), and leptin were significantly higher in MetS infertile patients (*P* < .001; *P* < .005; *P* < .001; *P* < .001; and *P* < .001, respectively). While T and high-density lipoprotein (HDL) were significantly lower in MetS infertile patients (*P* < .01, and *P* < .001, respectively). Leptin was correlated with sperm progressive motility (*R* = −0.312, *P* < .01), and normal morphology (*R* = −0.458, *P* < .001). Moreover, sperm concentration was correlated with FSH (*R* = −0.268, *P* < .001) and inhibin B (*R* = 0.401, *P* < .001), and normal morphology was correlated with HDL (*R* = 0.233, *P* < .001) and TG (*R* = −0.182, *P* < .01). In primary infertile patients, sperm normal morphology were found to be depressed and related to MetS. Leptin was increased in patients diagnosed with MetS and associated with semen parameters.

## 1. Introduction

Up to 12% of couples experience infertility, and the frequency of male factor infertility may be rising.^[1]^ Notably, a sufficient quantity of high-quality spermatozoa is required for male fertility. Numerous investigations have documented the drop in semen parameters.^[2]^ Over the previous 20 years, there appears to have been a decline in sperm concentration, total sperm count, sperm progressive motility, and normal morphology.^[3]^

Metabolic syndrome (MetS) has been suggested as a possible contributing cause to the deterioration in semen quality and a contributing factor to male infertility due to the rising prevalence of obesity and diabetes. A number of health conditions, such as abdominal obesity, elevated blood pressure, elevated blood sugar, elevated triglycerides, and decreased high-concentration lipoprotein cholesterol (HDL-C), are associated with MetS.^[4]^ Obesity turns into a serious health issue and is rising alarmingly globally.^[5]^ Obesity has been shown to have detrimental effects on male reproductive function in both clinical and experimental research. Spermatogenesis, sperm maturation, and capacitation disorders were intimately linked to abnormal lipid metabolism. Changes in plasma cholesterol levels may have an impact on reproductive health.^[6,7]^

Obesity turns into a serious health issue and is rising alarmingly globally.^[8]^ Obesity has been shown to have detrimental effects on male reproductive function in both clinical and experimental research.^[9]^ Through restraining appetite, promoting heat production, and enlarging energy expenditure, leptin acts as a crucial regulator in the long-term control of body weight.^[10]^ An elevated plasma leptin level is supposed to involve in the pathogenesis of MetS and its various components.^[11]^ Due to the activation of the hypothalamic-pituitary-adrenal axis, and/or stimulating the secreting of lipolytic hormones, high levels of leptin eventually leads to several constituents of MetS, such as dyslipidemia, abdominal obesity, and insulin resistance.^[12]^ Studies on both experimental animals and humans have pointed out leptin potential role in the regulation of reproduction functioning as a metabolic junction of nutritional status and fertility.^[13–15]^ Despite leptin well proven effects on fertility in female, it remains debatable how it plays a part in the complicated regulatory network affecting male reproduction.^[16,17]^

There is currently a lack of comprehensive study on whether the combined effect of obesity and metabolic syndrome on fertility is higher, despite the fact that they are connected and may each have an influence on fertility. The objective of this study was to assess the serum leptin levels in Chinese patients and determine the relationships between these levels and MetS and semen parameters in infertile patients.

## 2. Method

### 2.1. Patients

We evaluated 200 primary infertility patients and 30 healthy controls (HCs) at Gongli Hospital between March 1, 2020, and March 1, 2024. Diagnostic criteria for MetS: once patients presented with 3 or more of the following: waist circumference ≥ 102 cm; triglycerides (TG) ≥ 1.7 mmol/L, or have received medical treatment; high-density lipoprotein (HDL) < 1.03 mmol/L, or have received drug therapy; blood pressure ≥ 130/85 mm Hg, or have received medication; fasting blood glucose (FBG) ≥ 5.6 mmol/L, or have received medication. Exclusion criteria: Patients who had a genetic abnormality (such as karyotypic abnormalities and Y chromosome microdeletions), or were diagnosed as azoospermia (obstructive or nonobstructive azoospermia). The author is not allowed to access information that can identify individual participants during or after data collection. This study obtained approval from the Ethical Committee of Gongli Hospital.

### 2.2. Study design

Subgroups of patients were based on presence of MetS or not, which is defined by National Cholesterol Education Programme, Adult Treatment Panel III. Basic information such as age, height, weight, waist circumference, and blood pressure of the patients were collected. The testicular volume was measured by testicular ultrasonography. The fasting blood test was performed before 10:00am, and it includes follicle-stimulating hormone (FSH), luteinizing hormone (LH), prolactin (PRL), estradiol (E2), testosterone (T), free testosterone (fT), sex hormone binding globulin (SHBG), inhibin B, FBG, triglyceride (TG), high-density lipoprotein (HDL). Semen sample was collected by masturbation in our sampling room after 4 to 7 days of abstinence. The computer-assisted sperm quality analysis system and computer morphology analysis system were used to perform semen analysis and sperm morphology detection. The sperm concentration < 20 × 10^6^/mL, (A + B) grade sperm < 50% or A grade sperm < 25% are identified as abnormal according to the reference range established in our laboratory.

### 2.3. Instruments and reagents

Fasting glucose levels were detected by a glucose oxidase method (Aeroset Abbott, Rome, Italy). CHO, HDL, and TG levels were detected by the automated enzymatic colorimetric method (Aeroset Abbott). Sex hormones were measured with a heterogeneous competitive magnetic separation assay (Bayer Immuno 1 System; Bayer Corp., Tarrytown, NY, USA). Inh B was detected by an enzyme-linked immunosorbent assay (Beckman Coulter AMH Gen II ELISA, High Wycombe, UK). SHBG level was detected by a solid-phase chemiluminescent immunometric assay on Immulite 2000 (Medical Systems SpA, Genoa, Italy).

### 2.4. Statistical analysis

SPSS 24.0 (SPSS Inc., Chicago) was chosen to analyze data. Mean and SD were used to describe continuous variables and frequencies and percentages for categorical variables when patients’ demographic features are involved. Differences between these 2 kinds of variables were tested by the Student *t* test or Mann–Whitney *U* test and Chi-square test, respectively. The comparison among the parameters of the 3 groups were accomplished by 1-way analysis of variance or Kruskal–Wallis tests based on the distribution of the data and post hoc analyses were performed by Bonferroni or Temphane tests. Spearman correlation analysis was used to analyze the correlation. *P* < .05 was considered to be of statistical significance.

## 3. Results

### 3.1. Sociodemographic and clinical features of infertile patients with or without metabolic syndrome

The mean age of infertile patients with MetS was 32.13 ± 4.98 years, while it of infertile patients without MetS was 30.17 ± 3.89 years. The mean disease duration, testis volume of infertile patients with or without MetS were 1.23 ± 0.12, 1.55 ± 0.35 years; 14.45 ± 2.9, 13.53 ± 2.35 mL, respectively. There was no significant difference in these variables (*P* > .05). What’s more, no differences were discovered between groups in smoking habits. However, the difference in BMI was statistically significant between MetS and non-MetS infertile patients (28.28 ± 1.93 vs 23.99 ± 4.66, *P* < .05). Table 1 listed detail information about sociodemographic and clinical characteristics of the patients.

**Table 1:** Sociodemographic and clinical features of infertile patients with or without metabolic syndrome.

	Infertile patients with MetS n = 100	Infertile patients without MetS n = 100	*P* value
Age (yr, mean ± SD)	32.13 ± 4.98	30.17 ± 3.89	NS
Current smoker (%)	25.0	23.0	NS
BMI (kg/m^2^, mean ± SD)	28.28 ± 1.93	23.99 ± 4.66	0.001
Testis volume (mL, mean ± SD)	14.45 ± 2.9	13.53 ± 2.35	NS
Duration of infertility (yr, mean ± SD)	1.23 ± 0.12	1.55 ± 0.35	NS

Abbreviation: BMI = body mass index.

### 3.2. Comparison of infertile patients with or without metabolic syndrome

The comparison between the demographic features and biomarkers according to presence of MetS, age, disease course, testis volume, LH, PRL, E2, and inhibin B were similar (*P* > .05). BMI, glucose, triglyceride, FSH, and leptin were significantly higher in MetS infertile patients (*P* = .001; *P* = .005; *P* < .001; *P* = .001; and *P* < .001, respectively) (Tables 2 and 3). While T and HDL were significantly lower in MetS infertile patients (*P* = .01 and *P* = .001, respectively) (Table 2 and 3). No differences were detected between groups regarding semen volume, sperm concentration, progressive motility, and normal morphology (*P* > .05).

**Table 2:** Comparison of semen parameters, endocrine hormones, cholesterol and biochemical levels of infertile patients and healthy controls (variance analyses).

	Infertile patients with MetS n = 100	Infertile patients without MetS n = 100	Healthy controls n = 30	*P* value
Glucose (mg/dL)	5.24 ± 0.63	4.75 ± 0.67	4.67 ± 0.32	NS
HDL (mg/dL)	1.64 ± 0.37	2.26 ± 0.72	2.46 ± 0.67	NS
Triglyceride (mg/dL)	2.97 ± 1.05	1.38 ± 0.48	1.53 ± 0.52	NS
Leptin (ng/mL)	7.63 ± 1.68	4.82 ± 0.76	4.52 ± 0.52	NS
FSH (mUI/mL)	7.64 ± 3.12	5.78 ± 2.05	5.51 ± 2.12	NS
LH (mUI/mL)	4.12 ± 2.42	3.78 ± 1.63	3.56 ± 1.31	NS
PRL (ng/mL)	12.11 ± 4.16	13.78 ± 4.12	12.32 ± 3.78	NS
E2 (pg/mL)	36.86 ± 12.85	38.57 ± 13.56	38.65 ± 11.21	NS
T (ng/mL)	4.53 ± 1.69	5.17 ± 1.33	5.32 ± 1.47	NS
SHBG (nmol/L)	29.96 ± 4.14	30.67 ± 5.12	31.22 ± 4.23	NS
Inhibin B (pg/mL)	104.42 ± 39.21	128.78 ± 46.45	131.11 ± 51.31	NS
Semen volume (mL)	3.11 ± 1.19	2.99 ± 1.54	3.25 ± 1.17	NS
Sperm concentration (×10^6^/mL)	17.53 ± 12.54	22.50 ± 16.43	29.92 ± 15.78	<.001
Progressive motility (%)	22.34 ± 16.35	29.65 ± 24.56	60.34 ± 9.12	<.001
Normal morphology (%)	5.25 ± 1.19	7.12 ± 1.69	9.01 ± 1.23	<.001

Abbreviations: E2 = estradiol, FSH = follicle-stimulating hormone, HDL = high-density lipoprotein, LH = luteinizing hormone, PRL = prolactin, SHBG = sex hormone binding globulin, T = testosterone, TG = triglycerides.

**Table 3:** Comparison of semen parameters, endocrine hormones, cholesterol and biochemical levels of infertile patients and healthy controls (post hoc analysis).

	MetS (+)/MetS(−) *P* value	MetS (+)/healthy controls *P* value	MetS (−)/healthy controls *P* value
Glucose (mg/dL)	.005	.007	NS
HDL (mg/dL)	.001	<.001	NS
Triglyceride (mg/dL)	<.001	<.001	NS
Leptin (ng/mL)	<.001	<.001	NS
FSH (mUI/mL)	.001	<.001	NS
LH (mUI/mL)	NS	NS	NS
PRL (ng/mL)	NS	NS	NS
E2 (pg/mL)	NS	NS	NS
T (ng/mL)	.01	.032	NS
SHBG (nmol/L)	NS	NS	NS
Inhibin B (pg/mL)	NS	<.001	<.001
Semen volume (mL)	NS	NS	NS
Sperm concentration (×10^6^/mL)	NS	<.001	<.001
Progressive motility (%)	NS	<.001	<.001
Normal morphology (%)	NS	<.001	<.001

Abbreviations: E2 = estradiol, FSH = follicle-stimulating hormone, HDL = high-density lipoprotein, LH = luteinizing hormone, PRL = prolactin, SHBG = sex hormone binding globulin, T = testosterone, TG = triglycerides.

As shown in the additional analyses, leptin was correlated with sperm progressive motility (*R* = −0.312, *P* = .01), and normal morphology (*R* = −0.458, *P* < .001). Moreover, sperm concentration was correlated with FSH (*R* = −0.268, *P* < .001) and inhibin B (*R* = 0.401, *P* < .001), and normal morphology was correlated with HDL (*R* = 0.233, *P* = .001) and triglyceride (*R* = −0.182, *P* = .01) (Table 4).

**Table 4:** Correlation of clinical features, endocrine hormones, cholesterol and biochemical levels with semen parameters in infertile patients.

	Sperm concentration		Progressive motility		Normal morphology	
	*R*	*P* value	*R*	*P* value	*R*	*P* value
Age	―	NS	―	NS	―	NS
BMI	―	NS	―	NS	―	NS
Testis volume	―	NS	―	NS	―	NS
Glucose	―	NS	―	NS	―	NS
HDL	―	NS	―	NS	0.233	.001
Triglyceride	―	NS	―	NS	-0.182	.01
Leptin	―	NS	−0.312	.01	−0.458	<.001
FSH	−0.268	<.001	―	NS	―	NS
LH	―	NS	―	NS	―	NS
PRL	―	NS	―	NS	―	NS
E2	―	NS	―	NS	―	NS
T	―	NS	―	NS	―	NS
SHBG	―	NS	―	NS	―	NS
Inhibin B	0.401	<.001	―	NS	―	NS

Abbreviations: E2 = estradiol, FSH = follicle-stimulating hormone, HDL = high-density lipoprotein, LH = luteinizing hormone, PRL = prolactin, SHBG = sex hormone binding globulin, T = testosterone, TG = triglycerides.

### 3.3. Comparison of infertile patients with healthy controls

Tables 2 and 3 listed the variance and post hoc analyses and we discovered elevated leptin level in both MetS^+^ and MetS^−^ than the HCs (*P* < .001), and that inhibin B, sperm concentration, progressive motility, and normal morphology were higher in HCs than both MetS^+^ and MetS^−^ infertile patients (*P* < .001). FSH was higher while T was lower in MetS^+^ infertile patients than HCs (*P* < .001, *P* = .032), but they showed no significant difference between HCs and MetS^−^ infertile patients (*P* > .05).

## 4. Discussion

In this study, we assessed the relationships between serum biomarkers and semen parameters in patients with infertility, according to the presence of MetS comparing with the HCs, and the significance of serum leptin level in the correlation with semen quality. A major finding of our study was that leptin levels of MetS^+^ infertile patients were higher than the MetS^−^ infertile patients and HCs, suggesting that elevated serum leptin levels may be related with MetS and semen parameters in infertile patients.

As a hormone coded by obese gene, leptin is mainly secreted by adipocytes with a dual role of regulator in energy homeostasis and reproductive system. Obese gene deficiencies in humans and rats lead to various male reproductive abnormalities, including reduction in the weights of ventral prostate and testes, decreased amounts of sperms in the seminiferous tubules and smaller size of Leydig cells.^[18]^ Since researchers observed elevated plasma leptin levels in azoospermic patients compared to health controls and no relations were found between plasma leptin levels, FSH and LH, leptin is supposed to target directly on testis.^[19,20]^

We found that infertile patients with higher leptin levels had lower progressive motility, which is in accordance with other studies. Ahima et al described a negative signification between leptin and sperm motility.^[21]^ Glander et al found a negative correlation between leptin and the motility of human spermatozoa.^[22]^

Nevertheless, there are studies founding no relationship between seminal leptin concentration and spermiogram parameters.^[23]^ In our study, we also couldn’t find the relationship between leptin and sperm concentration, but we find the relationship between leptin and sperm morphology. Hence it still remains unclear how leptin participates in male reproductive function. Another main finding in our study was that TG and HDL levels of infertile patients were significantly related to sperm morphology, implying that MetS^+^ may have a bad influence on sperm morphology, and this is in accordance with other studies which have reported the decreased normal morphology in infertile patients with MetS.^[24,25]^

In reality, the complicated procedure of production and maturation of sperms are strictly and precisely regulated.^[26]^ However, MetS can alter the necessary microenvironment required by such processes. In general, MetS, especially the excessive visceral adiposity showed in our results, can change hormone levels and promote chronic inflammation and oxidative stress in reproductive tract, and accumulated fat leads to a warmer environment within scrotum^[8,27,28]^ In conclusion, the subsequent alteration of the vital conditions in testes and epididymis can disturb the processes of spermatogenesis and sperm maturation.

Dysregulation of lipid metabolism, defined by elevated triglycerides and/or low HDL, is another key component of MetS. The link between lipid homeostasis and fertility was found in patients who suffered from hyperlipidemia or MetS. Kinds of saturated and unsaturated fatty acids make up the framework of cell membrane of spermatozoon and count for the sperm function, including sperm motility, viability and fertility. MetS, usually accompanied by chronic inflammation, brings about fast metabolism and stimulates production of ROS within testicular tissue, reproductive tract and semen.^[27]^ The unsaturated fatty acids become fragile when exposed to ROS and then lipid peroxidation takes place.

As a result, alterations in the membranous fatty acid may be 1 of the reasons why sperm quality is damaged. At the same time, cholesterol is a major component as well, being various during sperm maturation and capacitation. It is supposed that dysregulation of cholesterol in sperm are related to abnormal morphology, impaired motility and premature acrosome reaction.^[29]^ Overall, those mechanisms, including altered lipid constituents and cholesterol, accelerated ROS formation and DNA injury, which may impair spermatogenesis and sperm maturation and finally lead to male subfertility or infertility.

We have known that leptin targeting a great many organs and tissues. Surprisingly, how leptin exerts effects centrally and peripherally exhibits similarity, to some extent, to the manner of other hormones such as insulin.^[30]^ Leptin also participates in the stimulation of the complex neuroendocrine regulation of the energy homeostasis and fertility. Some studies showed that supplement of exogenous leptin to mammals with malfunction in synthesis of endogenous leptin will reinforce the control of metabolism and restore reproductive function.^[31–33]^ All of the above provide evidence for the opinion that leptin, within the physiological concentration, involves in the regulation of metabolic and vascular homeostasis. On the contrary, long-term high concentrations of leptin will cause metabolic dysregulation.^[34]^ Excessive leptin can inhibit the activity of acetyl coenzyme A carboxylase, resulting in increased fatty acid oxidation in muscle.^[35]^ Leptin pro-inflammatory function seems to result in endothelial dysfunction and insulin resistance.^[36,37]^ Based on the above evidence, we could theorize that MetS and leptin can not only affect semen quality alone, but also can interact with each other, aggravating the impact on semen quality.

Our study was limited by the relatively small sample capacity resulting in the indefiniteness of the conclusion. In addition, because of the variety of causes leading to male infertility and the differences in genetic background, lifestyle and living environment, our results might have some confounding errors.

In conclusion, in infertile patients, sperm normal morphology were depressed and in relation to MetS indicating the significance of looking into MetS in patients with infertility. Certain biomarkers in infertile patients, especially leptin, elevated in those presented with MetS and were associated with semen parameters. Therefore, the comprehensive treatment of obesity and MetS may be beneficial to improve semen qualities in infertile patients.

## Author contributions

**Conceptualization:** Xin Song.

**Data curation:** Xin Song.

**Formal analysis:** Deping Dong.

**Investigation:** Xin Song.

**Methodology:** Xin Song.

**Project administration:** Zhen Dong.

**Resources:** Zhen Dong.

**Software:** Zhen Dong.

**Supervision:** Zhikang Cai.

**Validation:** Xin Song.

**Visualization:** Deping Dong, Zhikang Cai.

**Writing – original draft:** Xin Song.

**Writing – review & editing:** Deping Dong, Zhikang Cai.
